# Liking, preference and practical implications of protein and energy enriched in-between-meals designed for elderly people

**DOI:** 10.29219/fnr.v65.5635

**Published:** 2021-02-15

**Authors:** Karin Wendin, Maria Biörklund-Helgesson, Kristina Andersson-Stefanovic, Anders Lareke, Olof Böök, Christina Skjöldebrand

**Affiliations:** 1 Department of Food and Meal Science, Kristianstad University, Kristianstad, Sweden; 2 Department of Food Science, University of Copenhagen, Frederiksberg C, Denmark; 3 Community of Ystad, Ystad, Sweden; 4 En God Granne AB, Ystad, Sweden; 5 Aventure AB, Lund, Sweden; 6 CFB Creative Future Business AB, Saltsjö-Boo, Sweden

**Keywords:** elderly, care staff, in-between meals, protein and energy enrichment, sensory attributes, nutrition

## Abstract

**Background:**

An adequate dietary intake, especially of protein and energy, is important for maintaining health among elderly people, especially those in care homes. One strategy to ensure nutritional intake is to customise attractive products through enrichment to match the needs of elderly people in care homes.

**Objective:**

To evaluate liking and practical aspects of protein and energy enriched in-between meals designed for elderly people in care homes through the use of quantitative and qualitative assessments.

**Design:**

A broad range of energy and protein enriched in-between meals, including both savoury and sweet products, were included. The products were evaluated by a consumer test and a focus group discussion with elderly respondents. The products were also evaluated by a second focus group discussion with care staff.

**Results:**

The most liked products were ice cream and cheesecake. All products achieved high scores for appearance, taste/flavour and texture. No product included in the study was extremely disliked. However, the least liked product was tomato soup, which scored above the middle of the scale except for texture. It was clear from the focus group discussions that a colourful appearance, small portion size and texture were of primary importance. The temperature had an impact on liking and swallowability.

**Discussion:**

Most products were perceived by the elderly participants as appealing and tasting good, and possible to include in a daily diet. It was clear that the colours of the foods were of primary importance. In line with other studies, it was found that highly liked in-between meals were frozen, cold and sweet. These products were also easy to swallow.

**Conclusions:**

It is possible to produce highly liked energy and protein enriched in-between meal products designed for elderly people. The temperature had a great impact on the liking of texture, taste and flavour. In-between meals should preferably be colourful and have a small portion size.

## Popular scientific summary

- Energy and protein enriched in-between-meals may be used for maintaining health among elderly people.
- Frozen, cold and sweet in-between meal products are among the most liked.
- The temperature needs to be right for the product to reach a high liking of texture, taste and flavour.
- Preferred in-between-meals should be colourful and have a small portion size.

The global population aged 60 years and older is expected to rise from 900 million in 2018 to a total of 2 billion by 2050. Today, 125 million people are 80 years or older (1). The number of Europeans aged 65+ years will increase by approximately 11% between 2018 and 2100 (2). Many elderly people are living a healthy, active and independent life (3) and one of the key factors for maintaining health and quality of life is an adequate dietary intake. However, changes in nutritional needs with ageing have to be taken into account. The needs for an increased protein intake as well as sufficient energy intake are of primary importance (4, 5). Changes in nutritional needs, together with an increased risk of disease and disability, make older adults vulnerable to disease-related malnutrition (DRM). Disease-related malnutrition leads to a weaker immune system, bone and muscle loss, increased mortality and poor quality of life (6–8). People suffering from DRM place a high economic burden on healthcare services (9, 10). It is, however, possible to proactively counteract DRM through adequate food intakes, for example by using foods containing high amounts of high-quality proteins and energy (11–13).

Poor appetite is often associated with poor nutritional intake (12). One common reason for this is dysphagia, that is chewing and swallowing disorders, where texture modified foods are necessity to guarantee an adequate nutritional intake (14–16). Another reason for poor nutritional intake is changes in the sensory system. Dysfunction of the senses increases with age and causes elevated thresholds for taste and smell and a decreased ability to identify tastes and odours. Hence, the older person does not recognise the food experience, which may be a contributing cause of loss of appetite (17–20). Findings suggest that the age-related decline in olfactory sensitivity is not uniform but rather odour specific (21). Inter-individual variation in the loss of perception is also significant (22).

One strategy to increase nutritional intake, especially among elderly people in care homes, is to customise attractive products in accordance with the needs of the older persons (23). It has been shown that protein enrichment of regular food products can significantly increase the daily protein intake (24). It is also well known that large portions of food may be overwhelming, which discourages intake (25). The customised products must, therefore, be offered in small servings and be dense in nutrients and energy (26). The formulation and processing of such food is not straightforward since sufficient energy and nutrients have to be provided in a limited volume. Protein enrichment often affects the taste, flavour and texture of foods, which may be a problem. The addition of protein may result in drier, crumblier and overly bitter products; however, different types of proteins affect products differently (27). The addition of fat also affects sensory characteristics, for example baked products may become flat and moist with a smooth texture and have a fatty mouthfeel. In the co-addition of fat and protein, the effect of added protein appears to dominate the effects of added fat (28). Moreover, it is recommended that protein enrichment is based on carriers that are already commonly consumed by older adults. The levels of enrichment should also not result in the characteristics of the food product being altered to such a degree that the product is no longer acceptable (28, 29). Recent research has shown that flavour and sensory-based ranking of in-between meals opens the possibility of designing new enriched meals for older adults by choosing those that are most liked by the elderly in care homes (30). A way to design meals for undernourished elderly people in care homes could be to offer them these nutritious in-between meals in order to be able to serve what is both liked and needed (30).

This research study investigates the potential of protein and energy enriched in-between meals designed for elderly people residing in care homes in Sweden, based upon a broad range of common everyday food items. The products were designed using an innovative process where ordinary food products were adapted to the specific needs of the target group (31, 32).

## Aim

The aim of the study was to evaluate liking and practical aspects of protein and energy-enriched in-between meals designed for elderly people in care homes through the use of quantitative and qualitative assessments.

## Material and methods

### Food products

The study included a broad range of food products prepared as in-between meals, including both savoury and sweet products. The in-between meals were enriched with energy and protein using ingredients from sources that occurred naturally in the products. The goal was to attractively develop tasty and enriched in-between meal products adapted to the needs of elderly people, while retaining its similarity to the original food products as far as possible. Products included in the study were required to be served as an in-between meal with a small portion size (100–200 g), a minimum protein content of 5 g per portion and a minimum energy content of 50 kcal per 100 g. The enriched in-between meal products were developed at Swedish small and medium-sized enterprises (SME) within the food industry. The companies were: Aventure AB, which develops and produces innovative and healthy products; Alvestaglass AB, that produces ice cream; Kronägg AB, producer of eggs and egg products; Ostkaksbageriet i Vrigstad AB, which produces traditional, Swedish cheese cake; Lindvalls Chark AB, that produces charcuteries and finally, Fazer AB, which is a bakery company. The products connected to the manufacturing company are presented in Table 1.

**Table 1:** In-between meals included in the study. Producer as well as energy and protein contents are shown

Product	Producer	Serving size	Energy	Protein	Energy	Protein
			kcal per serving	gram per serving	per 100 g (kcal)	per 100 g (g)
Oat-based drink Strawberry flavour	Aventure	200 mL	274	10	137	5
Oat-based drink Exotic flavour	Aventure	200 mL	274	10	137	5
Oat-based soup Tomato flavour	Aventure	200 mL	274	10	137	5
Ice cream Vanilla flavour	Alvesta glass	200 mL	412	20	206	10
Egg-based ice cream Lemon flavour	Kronägg	200 mL	420	22	210	11
Egg-based drink Chocolate flavour	Kronägg	200 mL	240	22	120	11
Swedish Cheesecake	Ostkaksbageriet i Vrigstad	100 g	260	12	260	12
Cocktail Sausages	Lindvalls Chark	100 g	270	12	270	12
Bread roll	Fazer	140 g	240	6	240	10

The in-between meal products were transported directly from the food company to a care home in the city of Ystad, Sweden, where they were stored for approximately 3–5 days until the hedonic tests were carried out. The storage took place either in a fridge (5°C) or a freezer (−22°C).

The test samples of the in-between meals were smaller in size than the serving size. They were prepared and served either on a 15 cm diameter paper plate (cheesecake, cocktail sausage, bread roll), in a 40 mL plastic cup (oat-based drinks, oat-based soup, egg-based drink) or in a 30 mL plastic bowl (ice creams). The drinks were served chilled directly from the fridge and the ice creams were served from the freezer after thawing for 10 min at an ambient temperature (approximately 22°C). The soup, cheesecake and sausage were heated and served at approximately 60°C.

### Subjects

Elderly people living in a care home and free-living elderly people with home care connected to the same care home in the city of Ystad, Sweden were invited to participate in a hedonic sensory test of in-between meal products and a focus group discussion concerning in-between meals. Six women and four men from three different wards at the care home and from free-living accommodation accepted the invitation; all were aged 80 years or older and cognitively undiminished.

Prior to the test, the elderly participants had the opportunity to test and eat the in-between meal products during an 8 week period and were therefore already familiar with the test products.

Care staff was invited to participate in a focus group discussion separate from the focus group with the elderly participants. Ten women aged 51–64 years and working at the care home, or in connected home care accepted the invitation.

The care staff were acquainted with the in-between meal products having served them to the elderly people during the 8 week period. Their task was to consider the food from the perspective of the elderly, their own experience of serving meals to the elderly, and what they perceived as important product requirements.

The Swedish Ethics Review Act ‘applies to research carried out in Sweden if the research includes the processing of sensitive personal data’. This study includes questions about food opinions which, according to the Data Protection Ordinance, are not classified as sensitive personal data. According to General Data Protection Regulation (GDPR), no responses to any of the questionnaires used in this study include information that can be traced to, or used to identify any individual. All participants received written and oral information about the hedonic test and focus group, and gave informed consent to participate. All subjects were familiar with the in-between meals included since these had been served as alternatives to the previously offered in-between meals during a period of 8 weeks prior to the hedonic test and focus group discussions.

### Hedonic test

The test measured the degree of liking of the in-between meal products using the 9-Point Hedonic Scale ranging from ‘dislike extremely’ (=1) to ‘like extremely’ (=9). The participants filled out a paper questionnaire for each test sample. Each sample was assessed with respect to:

- Appearance
- Taste/Flavour
- Texture

The products were tested one by one in the same order by all participants with a short break between each serving.

The hedonic test took place during the day time and was performed as a centrally located test (CLT), which is in a meeting place with large table space for all participants.

### Statistical evaluation

The hedonic data was evaluated using a multiple comparison test by one-way Analysis of Variance (ANOVAs) in conjunction with Tukey’s Post-Hoc Tests to compare included samples. The significance level was set to *P* < 0.05, SPSS (Version 23, IBM, New York, NY, USA).

### Focus group discussions

Two separate focus group discussions were undertaken in correlation to the hedonic product testing. One focus group discussion was held with the same elderly participants as in the hedonic test and one with care staff participants. The discussions in each group were moderated by persons with long experience in the area of food for the elderly. The discussions were recorded using mobile phones (Apple IPhone) and notes were taken by an assistant in each group. The same semi-structured interview guide was used in both groups. The guide was organised as follows:

- Food appearance
  - How should a food product look/appear in order to be liked?
  - How should a food product look/appear in order to be disliked?
  - Food taste and flavor
    - How should a food product taste in order to be liked?
    - How should a food product taste in order to be disliked?
    - Food texture
      - What texture should a food product have in order to be liked?
      - What texture should a food product have in order to be disliked?
      - Food serving

- How should a food product be served in order to be liked?
- How should a food product be served in order to be disliked?
- Food packaging
  - How should a food product be packaged in order to be liked?
  - How should a food product be packaged in order to be disliked?
  - The in-between meal products
    - Advantages and disadvantages?
    - Could the products be improved? How?

It should be noted that the elderly participants had already tasted the in-between meal products since the care staff had previously served the products to the elderly. Depending on the discussion, the moderators asked follow-up questions to gain further insight into the enriched in-between meals for elderly people.

### Qualitative evaluation

The recordings from the focus group discussions (*N* = 10 elderly and *N* = 10 care staff) were transcribed verbatim and the analysis of the qualitative data was based on content analysis with pre-defined main themes. The evaluation was based on systematically sorting the outcomes from the focus groups into the different themes.

## Results

### Hedonic test

The results showed that the in-between meal products with the highest liking scores were ice cream, both vanilla and lemon flavours, and Swedish cheesecake (Table 2). These three in-between meals scored seven or higher (mean value) of all the evaluated attributes appearance, taste/flavour and texture. None of the samples in the study was extremely disliked, although the tomato flavoured, oat-based soup was the least liked; however, except for texture, it scored above the middle of the scale.

**Table 2:** Hedonic test of the in-between meal product samples

Product	Appearance (*M* ± SD)	Taste/flavour (*M* ± SD)	Texture (*M* ± SD)
Oat-based drink Strawberry flavour	7.3 ± 0.8	7.0 ± 1.6	6.1 ± 2.5
Oat-based drink Exotic flavour	6.0 ± 1.9	6.5 ± 1.8	6.5 ± 2.1
Oat-based soup Tomato flavour	6.0 ± 1.1	5.3 ± 2.0	4.7 ± 1.5^a^
Ice cream Vanilla flavour	7.0 ± 1.5	7.8 ± 1.0	7.8 ± 1.0^b^
Egg-based ice cream Lemon flavour	7.0 ± 1.5	7.8 ± 1.0	7.8 ± 1.0^b^
Egg-based drink Chocolate flavour	7.5 ± 0.9	6.8 ± 2.0	7.0 ± 1.9
Swedish cheesecake	7.0 ± 0.0	7.0 ± 1.4	7.0 ± 0.0
Cocktail sausages	6.0 ± 2.1	6.1 ± 2.5	6.7 ± 2.1
Bread roll	7.7 ± 1.0	7.0 ± 1.3	6.0 ± 1.1

Note: Scale from 1 (dislike extremely) to 9 (like extremely). The different letters indicate a significant difference, which is also indicated by bold text.

From Table 2 it can be seen that the only significant difference between the in-between meal products was between the likings of texture, where the ice creams were significantly more liked than the tomato flavoured oat-based soup. It can further be noted high scores, however not significant, for the appearance of the strawberry and chocolate drinks, also the appearance of the bread roll scored high. Due to taste and flavour both ice creams, vanilla and lemon, scored high.

### Focus group – Elderly people

#### Food appearance

The appearance of a food was discussed to create expectations of its qualities and how it should taste when eaten. The participants in the focus group agreed that in order for it to be liked, the product should have an attractive appearance – it should look appetising! Colour and size were said to be of greater importance than the shape. However, well-balanced combinations of colour, size and shape were the most important.

‘There should be a touch of colour and the way the food is arranged on the plate has great impact on liking’.

Further, there was an agreement that if vegetables are missing in a meal, the meal is regarded as dull and colourless. Dull and colourless foods were disliked.

#### Food taste/flavour

Variation in the tastes and flavours of the products were of utmost importance. The food should be low in spiciness; however, spices should be available for anyone who would like to make the food spicier. The group agreed however, that too spicy foods are disliked. A majority of the group participants also meant that the temperature of the food has to be right for the product, and has a great impact on the perceived taste and flavour.

The taste of a new food eaten for the first time will decide whether or not the specific food will be eaten again. The group meant that if the experience is bad or if expectations are not fulfilled, the food will probably be rejected if served again. It was clear that the individuals in the group preferred different foods and different tastes and flavours. One of the participants made it very clear by the following statement:

‘Liking or disliking a specific food or a specific taste is something very personal’.

#### Food texture

All persons in the group agreed that the texture had a great impact on product liking. Further, the discussion revealed that there are different expectations for different products and these expectations have to be fulfilled for the product to be liked. The temperature impacts the texture; for example, the texture of the tomato flavoured, oat-based soup was better liked if served warm rather than cold.

The dental status of the elderly was discussed and found important for the liking of different textures. For example, chewiness is important in vegetables. The texture of carrots should give some chewing resistance but, if included as part of a meal, the texture of the carrots should go with the specific meal. Toughness was disliked by all and it was mentioned that bread can be too tough. Graininess was also a texture that was disliked.

#### Food serving

A large majority of the elderly in the focus group was of the opinion that a meal should be served on a plate, the foods should be arranged carefully and the colours are an important factor for liking the meal. It was found that casseroles, pans and pots on the table were disliked. The vast majority of the participants wanted the foods to be served on a plate. Proper cutlery was preferred, and eating with the fingers was disliked.

Cleanliness and hygiene were said to be important, especially when eating together and sharing a meal.

The preferred time for serving the evening meal is around 18.30. In-between meals are preferred for consumption later in the evening. However, in-between meals could be served at any time during the day.

#### Food packaging

The packaging size was very important, that is the portion size should be correct. Large packaging was disliked. Moreover, packaging which is both easy to open and reseal is liked. The appearance of the packaging has to be connected to the food product inside, be informative and look neat. Glass packaging is disliked.

#### The in-between meal products

The portion sizes served were rated very good for all products. The names of the products were seen to give expectations of how the product was perceived and were therefore important for liking. Some respondents commented: *‘You can feel that the products are healthy’, ‘These products had a nice taste/flavour and had an attractive appearance’* and *‘I really hope that we can have these products in the future’*.

Overall impressions for each product, and agreed upon by all or an overwhelming number of the focus group are given in Table 3. It can be noted that the vanilla-flavoured ice cream which scored high in the hedonic test, could be improved by a more intense flavour. The texture of the lemon-flavoured ice cream scored high in the hedonic text and is considered as especially good when the ice cream starts to thaw.

**Table 3:** Comments on the in-between meal products from the focus group discussions with elderly people and care staff

Product	Elderly people	Care staff
Oat-based drink Strawberry flavour	Good taste/flavour in the beginning but after a while the strawberry flavour became acidic.	Very good texture when swallowing medicine.
Oat-based drink Exotic flavour	Very good taste/flavour.	Very good texture when swallowing medicine.
Oat-based soup Tomato flavour	Good if served hot. The texture is gritty when cold. The quality seems to differ between servings.	The colour was too pale. Too low in tomato flavour. The texture was too thick.
Ice cream Vanilla flavour	Very good, however the vanilla flavour could be improved by being more intense.	Appearance and texture were good.
Egg-based ice cream Lemon flavour	Very good, the texture is especially good if the ice cream has begun to thaw. The flavour is very good.	Appearance and texture were good.
Egg-based drink Chocolate flavour	Very good flavour. The texture is very good and the drink is easy to swallow.	Very good texture when swallowing medicine
Swedish cheesecake	The taste/flavour is very good if served warm. If warm, the tastes and flavours are more intense.	Good texture!
Cocktail sausages	Good!	Good texture!
Bread roll	Nice texture and shape. Especially liked when served with cheese.	Good texture!

Note: The given comments were agreed upon by all or a large majority of the participants in the focus groups.

### Focus group – Care staff

#### Food appearance

The care staff all agreed that food products have to be appealing at first sight and they should be colourful. The care staff said that many of the elderly people have poor eyesight and if the food is colourful it is easier to see. It is important that the colour is identical to the product, from which it should be possible to identify the product. The colour was said to be a very important indicator for liking and to identify what kind of product you are going to eat. When serving a meal, it should be possible to distinguish the different products on the plate. If a meal or a product is disliked due to appearance, the care staff had to persuade the elderly to taste it. One of the participating care staff concluded that:

‘If the elderly people dislike what they see, you can encourage them to taste it – that the food might anyway taste good!’

The elderly people sometimes face the problem of too much food on the plate, which overwhelms them and causes them to lose their appetite. However, it was discussed that it may be hard to decide what the right amount of food could be, since it differs from person to persons. The care staff could ask the elderly person when serving the food how much that person wants to eat.

#### Food taste/flavour

It could be concluded that the food should not be too spicy or too sweet. In this specific care home, many of the elderly persons were 90 years and older, and they did not like spices such as chili or too much pepper. Further, very few elderly people in this care home liked to eat very sweet foods. Spices were always served separately on the table and anyone could add extra spices to his or her food if they want. The elderly were said to like eating traditional, home-cooked food, for example brown beans, pea soup or other types of old-fashioned Swedish food.

#### Food texture

All of the care staff focus group had experienced that for many elderly, slices or large chunks of meat may be a problem; however, this was said to be dependent on the type and quality of the meat. On the one hand the elderly wanted real meat, for example cutlets on the bone and spareribs were popular. These can be difficult to chew but are still highly liked and the elderly can cut the meat into pieces and eat them with a sauce. On the other hand, it should be noted that elderly people prefer foods that are easy to chew. Most of the elderly like minced meat, especially those who need a special diet. Moreover, almost all elderly in this specific care home liked to eat dairy products and mousses, and these products work well from the perspective of texture.

#### Food serving

When serving a meal on a plate, each food component should be placed separately and be clearly visible. As mentioned above, colours were found to be very important. The use of a tablecloth may improve the impression of colour.

Sometimes the food in this elderly home is served as a buffet, where everyone either serves themselves or has assistance from the care staff, if needed. Some of the elderly people do not think they are able to eat or serve themselves well enough. However, they like to be sitting next to each other around a large table. Most of the care staff thought it would be a good idea if the elderly people and the staff could eat together, that would make it a more family-like and homely situation.

#### Food packaging

When discussing food packaging, the group meant that they should not be too big, especially when it comes to in-between meals where portion sizes are small and the elderly people have the option to choose between different products. This would lead to excessive waste since leftover food in the packaging is often not eaten. The packaging should not exceed 5 dL and it is important that it is easily resealable. The possibility of resealing the package was said to be very important to avoid spoilage of food, for example due to mould. The care staff also mentioned that, if a package has been opened and not resealed, it will not be used again since the food is no longer safe.

The discussion continued about whether it would be better to use one-portion-sized packages rather than large-sized packages to avoid food waste. Further, there should be clear and easy-to-read information on the package such as best-before date, content description and nutritional values. Information about storage before and after opening of the packages would also be good. Easily available information on how to eat and serve the product is needed. They mentioned that the most important thing was that the packaging should be easy to open and reseal.

#### The in-between meal products

The oats- and egg-based drinks were really good to drink when taking medication. It was so much easier for the elderly people to swallow their medications with these drinks compared to water. The texture worked well in all products. Products with a regionally specific taste profile were normally preferred by the elderly people.

Overall impressions for each product which were agreed upon by all or an overwhelming number of the focus group are given in Table 3.

## Discussion

The intake of food is highly connected to health and the in-between meal products in this study were considered to be healthy by the participants, especially in the focus groups with the elderly people. The in-between meal products enriched with energy and protein were chosen to cover a broad range of everyday foods, from savoury to sweet. The producers of the foods had great influence on the types of foods included and how to enrich, with the requirement that the products should have a minimum protein content of 5 g per portion and a minimum energy content of 50 kcal per 100 g. The product development was based on earlier work, where ordinary food products were adapted to the target group (31, 32). More or less, all of the in-between meal products were perceived by the elderly participants as appealing and tasting good, and they wanted to include them in their daily diet, indicating that the sensory properties of the in-between meals were highly acceptable. This is in line with earlier studies (23, 30) also showing that sensory properties are of greatest importance in order to fulfil energy and nutritional requirements among elderly people.

The results showed that the liking of the product appearance was high for products which had product-specific colours and shapes, for example the strawberry and chocolate drinks received high scores for their colour, and the bread for its shape. Other studies had similarly shown the impact of colour on liking, but also on perception of flavours (33), where it was seen that colours may trigger the perception of flavours (34).

In line with a study by Piqueras-Fiszman and Spence (35), the present study showed that a meal should be colourful. If the food on a plate appears dull and grey it is disliked at first sight. Moreover, the individual food components of a meal have to be visible. If the food is mixed and if it is hard to distinguish what the meal consists of, both intake and liking may decrease. Also, the size of a meal was discussed and considered to be a critical factor. If there is too much food on the plate, the person may feel overwhelmed and eat less of the food (25).

In parallel with colours, the tastes and flavours of in-between meals have to be product specific and fulfil expectations. The results showed that the flavours of the ice creams were highly appreciated. However, the elderly participants mentioned that the vanilla flavour of the ice cream was a little too weak, while the sour taste of the lemon-flavoured ice cream was very good. Sourness increases saliva secretion and may therefore aid the swallowing process (36) and thereby also impact liking. The ice cream texture was best liked when it had just started to thaw. Others have shown similar results; for example Okkels et al. (30) found that the most liked in-between meals were frozen, cold and sweet. Kälviäinen et al. (37) reported the importance of the texture of yoghurt. It is also well known that a cold temperature triggers the swallowing process and makes the product easier to swallow (38, 39), which may be another explanation for the high liking scores for ice cream.

The focus group with care staff reported that the flavour intensity of the tomato soup was too low. Both focus groups also mentioned that the liking of the tomato soup texture increased with higher temperature. Temperature has a high impact on flavour release and thereby, also on the perception of flavour (40, 41). The flavour intensity increased with the higher temperature due to increased flavour retention (42) and the texture became less gritty. The texture of all kind of foods is very important for both the perception and liking of specific foods. For elderly people, it is of even higher importance since chewing and swallowing difficulties (dysphagia) are very common (43). The problem of dysphagia and product-specific texture was pointed out in both focus groups. In this study, the texture of the ice creams was significantly more liked than the texture of the tomato soup.

Results from the focus group with care staff indicated that the texture of the chocolate drink was beneficial for the elderly people when taking oral medications. The drink was very easy to swallow, which was also reported in the focus group with the elderly participants. The slippery texture of the chocolate drink was caused by the egg protein ingredient (44).

It was pointed out in the discussions that there are large variations in individual preferences, and participants in both focus groups agreed that food should not be spicy, but spices should be easily available to add to their foods for those who wanted it.

It is interesting to note that the elderly people preferred being served food that was already plated, while the staff suggested that buffet serving was popular. The difference between the two focus groups results may be caused by the fact that the elderly people may sometimes find it hard to serve themselves due to tremor or weakness (45), while the staff may like to increase the independence of the elderly people and allow them to make independent choices. A more family-like situation may be possible if the staff were to eat together with the elderly people, as pointed out in the focus group with care staff. In earlier studies, so-called educational meals have been shown to be a trigger for a higher food intake, and the social dimension of the meal would also be of importance for well-being (46, 47).

The packaging of food should suit the product; one-portion sized package is preferred. If all the food is not taken out of the package, it should be possible to re-seal it. Both relevant and appealing information on the package are of importance.

From the results in this study it is promising that energy-rich and protein-enhanced in-between meals were highly liked. Both savoury and sweet products reached high liking scores. It has also shown the importance of the sensory aspects of the food. In line with Okkels et al. (30), it was clear that product specific colours, tastes and flavours, and textures were the driving factors for liking of the in-between-meal products. One way to design meals for elderly people could be to offer them nutritious in-between meals in order to be able to serve what is both liked and needed to prevent them becoming malnourished.

### Limitations

The number of participants in this study was low due to the small number of cognitively robust elderly persons living at the care home or free-living with home care. The results should, therefore, be regarded only as indications of how to further develop and manage enriched in-between meals for elderly people.

The serving of the samples of in-between meal food products was not randomised for practical reasons. The test was held at the care home and there was no equipment available to keep sample temperature uniform throughout the test.

## Conclusion

The results are of importance to guide producers of food designed for elderly people and for the health care in the ambition of avoiding malnutrition. It could be concluded that it is possible to produce highly-liked energy and protein enriched in-between meal products designed for elderly people. The temperature was shown to have great impact on the liking of texture, taste and flavour. Preferred in-between meals should be colourful and have a small portion size.

For future applications, a wider range of protein and energy enriched products should be produced and served as in-between meals.

## Conflict of interest and funding

This research study was financed by INCLuSilver, project no 731349 – H2020-INNOSUP-01-2016 within the European Union’s Horizon 2020 research and innovation program. There is no conflict of interests.

## References

[1] WHO. Ageing and health 2018. Available from: https://www.who.int/news-room/fact-sheets/detail/ageing-and-health [cited 07 October 2020].

[2] Eurostat. The EU’s population projected up to 2100 2020. Available from: https://ec.europa.eu/eurostat/web/products-eurostat-news/-/DDN-20190710-1 [cited 06 August 2020]

[3] Eurostat. Europe in figures: Eurostat yearbook 2011. Luxembourg: Office for Official Publications of the European Communities; 2011. Available from: https://ec.europa.eu/eurostat/documents/3217494/5728777/KS-HA-11-001-EN.PDF [cited 06 August 2020]

[4] Bauer J, Biolo G, Cederholm T, Cesari M, Cruz-Jentoft AJ, Morley JE, et al. Evidence-based recommendations for optimal dietary protein intake in older people: a position paper from the PROT-AGE Study Group. JAMDA 2013; 14: 8: 542–59. doi: 10.1016/j.jamda.2013.05.021 23867520

[5] Deutz NEP, Bauer JM, Barazzoni R, Biolo G, Boirie Y, Bosy-Westphal A, et al. Protein intake and exercise for optimal muscle function with aging: recommendations from the ESPEN Expert Group. Clin Nutr 2014; 33(6): 929–36. doi: 10.1016/j.clnu.2014.04.007 24814383 PMC4208946

[6] Liu L, Bopp MM, Roberson PK, Sullivan DH. Under nutrition and risk of mortality in elderly patients within 1 year of hospital discharge. J Gerontol A Biol Sci Med Sci 2002; 57A(11): M741–6. doi: 10.1093/gerona/57.11.M741 12403803

[7] Rasheed S, Woods RT. Malnutrition and quality of life in older people: a systematic review and meta-analysis. Ageing Res Rev 2013; 12(2): 561–6. doi: 10.1016/j.arr.2012.11.003 23228882

[8] Stratton RJ, Elia M. A review of reviews: a new look at the evidence for oral nutritional supplements in clinical practice. Clin Nutr Suppl 2007; 2(1): 5–23. doi: 10.1016/j.clnu.2007.04.004

[9] Amaral TF, Matos LC, Tavares MM, Subtil A, Martins R, Nazare M. The economic impact of disease-related malnutrition at hospital admission. Clin Nutr 2007; 26: 778–4. doi: 10.1016/j.clnu.2007.08.002 17936442

[10] Meijers JM, Halfens RJ, Wilson L, Schols JM. Estimating the costs associated with malnutrition in Dutch nursing homes. Clin Nutr 2012; 31(1): 65–8. doi: 10.1016/j.clnu.2011.08.009 21880401

[11] Schilp J, Wijnhoven HA, Deeg DJ, Visser M. Early determinants for the development of undernutrition in an older general population: longitudinal Aging Study Amsterdam. Br J Nutr 2011; 106(5): 708–17. doi: 10.1017/S0007114511000717 21450117

[12] Van der Meij BS, Wijnhoven HAH, Finlayson GS, Oosten BSH, Visser M. Specific food preferences of older adults with poor appetite. A forced-choice test conducted in various care settings. Appetite 2015; 90: 168–75. doi: 10.1016/j.appet.2015.03.011 25772198

[13] Mudge AM, Ross LJ, Young AM, Isenring EA, Banks MD. Helping understand nutritional gaps in the elderly (HUNGER): a prospective study of patient factors associated with inadequate nutritional intake in older medical inpatients. Clin Nutr 2011; 30: 320–5. doi: 10.1016/j.clnu.2010.12.007 21262553

[14] Rothenberg E, Ekman S, Bülow M, Möller K, Svantesson J, Wendin K. Texture-modified meat and carrot products for elderly people with dysphagia: preference in relation to health and oral status. Scand J Food Nutr 2007; 51(4): 141–7. doi: 10.3402/fnr.v51i4.1624

[15] Wendin K, Ekman S, Bülow M, Ekberg O, Johansson D, Rothenberg E, et al. Objective and quantitative definitions of food textures based on sensory and rheological methodology. Food Nutr Res 2010; 54: 5134. doi: 10.3402/fnr.v54i0.5I34 PMC2894641 20592965

[16] Aguilera JM, Park, DJ. Texture-modified foods for the elderly: status, technology and opportunities. Trends Food Sci Technol 2016; 57: 156–64. doi: 10.1016/j.tifs.2016.10.001

[17] Doty RL, Kamath V. The influences of age on olfaction: a review. Front Psychol 2014; 5(20): 1–20. doi: 10.3389/fpsyg.2014.00020 24570664 PMC3916729

[18] Duffy VB, Backstrand JR, Ferris AM. Olfactory dysfunction and related nutritional risk in free-living, elderly women. J Am Dietetic Assoc 1995; 95(8): 879–84. doi: 10.1016/S0002-8223(95)00244-8 7636078

[19] Croy I, Nordin S, Hummel T. Olfactory disorders and quality of life – an updated review. Chem Senses 2014; 39(3): 185–94. doi: 10.1093/chemse/bjt072 24429163

[20] Edfors E, Westergren A. Home-living elderly people’s views on food and meals. J Aging Res 2012; 761291: 1–9. doi: 10.1155/2012/761291 PMC3443996 22991667

[21] Seow, YX., Ong, PK., Huang D. Odor-specific loss of smell sensitivity with age as revealed by the specific sensitivity test. Chem Senses 2016; 41(6): 487–95. doi: 10.1093/chemse/bjw051 27001718

[22] Chen J, Engelen L. Food oral processing: fundamentals of eating and sensory perception. New Jersey: John Wiley & Sons, Wiley and Blackwell, USA; 2012.

[23] Rothenberg E, Wendin K. The ageing palate. Food Sci Technol 2015; 29(4): 2–5. doi: 10.13140/RG.2.1.4079.4968

[24] Stelten S, Dekker IM, Ronday EM, Thijs A, Boelsma E, Peppelenbos HW, et al. Protein-enriched regular products and their effect on protein intake in acute hospitalized older adults; a randomized controlled trial. Clin Nutr 2014; 34(3): 409–14. doi: 10.1016/j.clnu.2014.08.007 25179468

[25] Huffman GB. Evaluating and treating unintentional weight loss in the elderly. Am Fam Phys 2002; 65(4): 640–50. Available from: https://www.aafp.org/afp/2002/0215/p640.html [cited 06 August 2020] 11871682

[26] Nieuwenhuizen WF, Weenen H, Rigby P, Hetherington MM. Older adults and patients in need of nutritional support: review of current treatment options and factors influencing nutritional intake. Clin Nutr 2010; 29: 160–9. doi: 10.1016/j.clnu.2009.09.003 19828215

[27] Wendin K, Höglund E, Andersson M, Rothenberg E. Protein enriched foods and healthy ageing – effects of protein fortification on muffin characteristics. Agro Food Ind Hi Tech 2017; 28(5): 16–18.

[28] Höglund E, Albinsson B, Stuhr-Olsson G, Signäs M, Karlsson C, Rothenberg E, et al. Protein and energy enriched muffins designed for nutritional needs of older adults. Int J Food Sci Nutr 2017; 2(4): 555592. doi: 10.19080/NFSIJ.2017.02.555592

[29] Van der Zanden LDT, van Kleef E, Wijk RA, van Trijp HCM. Knowledge, perceptions and preferences of elderly regarding protein enriched functional food. Appetite 2014; 80: 16–22. doi: 10.1016/j.appet.2014.04.025 24798761

[30] Okkels SL, Saxosen M, Bügel S, Olsen A, Klausen TW, Beck AM. Acceptance of texture-modified in-between-meals among old adults with dysphagia. Clin Nutr ESPEN 2018; 25: 126–32. doi: 10.1016/j.clnesp.2018.03.119 29779807

[31] Hagermans S, Valerio L, Ombeline Viry J.Ice Cream snack for eldely patients. Lund: Master thesis Lund University; 2016.

[32] Kalouta K. Ostkaka (Swedish curd cake/Swedish cheese cake) – a new energy – dense product. Aventure, AB: Report Aventure. Lund; 2017.

[33] Zellner DA, Loss CR, Zearfoss J, Remolina S. It tastes as good as it looks! The effect of food presentation on liking for the flavor of food. Appetite 2014; 77: 31–5. doi: 10.1016/j.appet.2014.02.009 24589740

[34] Spence C. On the psychological impact of food colour. Flavour 2015; 4(1): 21. doi: 10.1186/s13411-015-0031-3

[35] Piqueras-Fiszman B, Spence C. Colour, pleasantness, and consumption behaviour within a meal. Appetite 2014; 75: 165–72. doi: 10.1016/j.appet.2014.01.004 24462488

[36] Bozorgi C, Holleufer C, Wendin K. Saliva secretion and swallowing – the impact of different types of food and drink on subsequent intake. Nutrients 2020; 12(1): 256. doi: 10.3390/nu12010256 PMC7019672 31963804

[37] Kälviäinen N, Roininen K, Tuorila H. The relative importance of texture, taste and aroma on a yogurt-type snack food preference in the young and the elderly. Food Qual Prefer 2003; 14(3): 177–86. doi: 10.1016/S0950-3293(02)00049-6

[38] Cola PC, Gatto AR, da Silva RG, Spadotto AA, Ribeiro PW, Schelp AO, et al. Taste and temperature in swallowing transit time after stroke. Cerebrovasc Dis Extra 2012; 2: 45–51. doi: 10.1159/000339888 23139681 PMC3493000

[39] Gatto AR, Cola PC, Silva RGD, Spadotto AA, Ribeiro PW, Schelp AO, et al. Sour taste and cold temperature in the oral phase of swallowing in patients after stroke. CoDAS 2013; 25(2): 163–7. doi: 10.1590/S2317-17822013000200012 24408246

[40] Kim JW, Samant SS, Seo Y, Seo, HS. Variation in saltiness perception of soup with respect to soup serving temperature and consumer dietary habits. Appetite 2015; 84: 73–8. doi: 10.1016/j.appet.2014.09.018 25265155

[41] Kähkönen P, Tuorila H, Hyvönen L. Dairy fat content and serving temperature as determinants of sensory and hedonic characteristics in cheese soup. Food Qual Prefer 1995; 6(2): 127–33. doi: 10.1016/0950-3293(95)98555-W

[42] Ventanas S, Mustonen S, Puolanne E, Tuorila H. Odour and flavour perception in flavoured model systems: influence of sodium chloride, umami compounds and serving temperature. Food Qual Prefer 2010; 21(5): 453–62. doi: 10.1016/j.foodqual.2009.11.003

[43] Su M, Zheng G, Chen Y, Xie H, Han W, Yang Q, et al. Clinical applications of IDDSI framework for texture recommendation for dysphagia patients. J Texture Stud 2018; 49(1): 2–10. doi: 10.1111/jtxs.12306 29052849

[44] Mine Y. Recent advances in egg protein functionality in the food system. Worlds Poult Sci J 2002; 58(1): 31–9. doi: 10.1079/WPS20020005

[45] Nyberg M, Olsson V, Örtman G, Pajalic Z, Andersson HS, Blücher A, et al. The meal as a performance: food and meal practices beyond health and nutrition. Ageing Soc 2018; 38(1): 83–107. doi: 10.1017/S0144686X16000945

[46] Herman CP. The social facilitation of eating or the facilitation of social eating? J Eat Disord 2017; 5(1): 1–5. doi: 10.1186/s40337-017-0146-2 28451432 PMC5406877

[47] Hetherington MM, Anderson AS, Norton GN, Newson L. Situational effects on meal intake: a comparison of eating alone and eating with others. Physiol Behav 2006; 88(4–5): 498–505. doi: 10.1016/j.physbeh.2006.04.025 16757007

